# Supplementary material for: 2-Pyridylmetallocenes, Part IX. Sulphur-Substituted 2-Pyridylferrocene: Synthesis and Reactivity towards Pt(II) and Hg(II)
Source: Molecules. 2024 Oct 15;29(20):4884. doi: 10.3390/molecules29204884 (PMC11510587; doi:10.3390/molecules29204884)
Supplement: Supplementary file 1 [file molecules-29-04884-s001.zip › molecules-3251339-supplementary.pdf]

Supplementary Information

2-Pyridylmetallocenes, Part IX. Sulphur-substituted 2-pyridylferrocene: Synthesis and reactivity towards Pt(II) and Hg(II)

*Stefan Weigand[1], and Karlheinz Sünkel **

Table of Contents

1. TABLES 2

2. NMR Spectra 3

Figure S 1: ^{1}H NMR spectrum ($CDCl_3$, 400 MHz) of compound **3** 3
Figure S 2 $^{13}C\{^{1}H\}$ NMR spectrum ($CDCl_3$, 101 MHz) of compound **3** 3
Figure S 3 ^{1}H NMR spectrum ($CDCl_3$, 270 MHz) of compound **5** 4
Figure S 4 $^{13}C\{^{1}H\}$ NMR spectrum ($CDCl_3$, 68 MHz) of compound **5** 4
Figure S 5 ^{1}H NMR spectrum ($CDCl_3$, 270 MHz) of compound **4** 5
Figure S 6 $^{13}C\{^{1}H\}$ NMR spectrum ($CDCl_3$, 68 MHz) of compound **4** 5
Figure S 7 ^{1}H NMR spectrum (CD_2Cl_2, 270 MHz) of compound **6** 6
Figure S 8 $^{13}C\{^{1}H\}$ NMR spectrum (CD_2Cl_2, 68 MHz) of compound **6** 6
Figure S 9 ^{1}H NMR spectrum (CD_2Cl_2, 270 MHz) of compound **7** 7
Figure S 10 $^{13}C\{^{1}H\}$ NMR spectrum (CD_2Cl_2, 68 MHz) of compound **7** 7

1. TABLES

Table S 1: Experimental Details of the Crystal Structure Determinations

Compound	**6**	**7**
Empirical formula	$C_{16}H_{15}Cl_2FeNPtS{\cdot}CH_2Cl_2$	$C_{16}H_{14}ClFeHgNS$
Formula weight	660.11	544.23
Temperature [K]	173(3)	173(2)
Crystal system	Trigonal	Triclinic
Space group	$P\,3_2$	*P* -1
Unit cell dimensions		
a [Å]	9.3525(4)	10.0318(7)
b	9.3525(4)	12.6529(10)
c	19.7170(8)	12.8460(10)
α [°]	90	87.834(6)
β	90	81.110(6)
γ	120	85.368(6)
V [$Å^3$]	1493.57(14)	1605.2(2)
Z	3	4
ρ_{calc} [g/cm^3]	2.202	2.252
μ [mm^{-1}]	8.388	10.743
F(000)	942	1024
Crystal size [mm^3]	0.183 x 0.175 x 0.091	0.280 x 0.120 x 0.100
Θ range [°]	4.358 – 26.297.	4.189 – 26.305.
Index ranges	-11≤h≤11, -11≤k≤11, -24≤l≤24	-8≤h≤12, -14≤k≤15, -15≤l≤16
Reflections collected	11245	8889
Independent reflections [R_{int}]	4036 [0.0510]	6432 [0.0789]
Absorption correction	Semi-empirical from equivalents	Semi-empirical from equivalents
T_{max}/T_{min}	1.0000 / 0.7129	1.0000 / 0.3347
Data / restraints / parameters	4036 / 1 / 228	6432 / 0 / 392
GOOF	1.010	1.035
R1/ wR2 [I>2σ(I)]	0.0392/ 0.0902	0.0495/ 0.1158
R1/ wR2 (all data)	0.0427/ 0.0913	0.0622/ 0.1299
Absolute structure parameter	-0.078(15)	N/A
Δρ (max/min) [e$Å^{-3}$]	1.719 / -0.697	2.812 and -2.642 $^{-3}$

2. NMR Spectra

Identified residual solvent impurities are marked: M: methylene chloride; W: water; EA: ethyl acetate; S: silicon grease

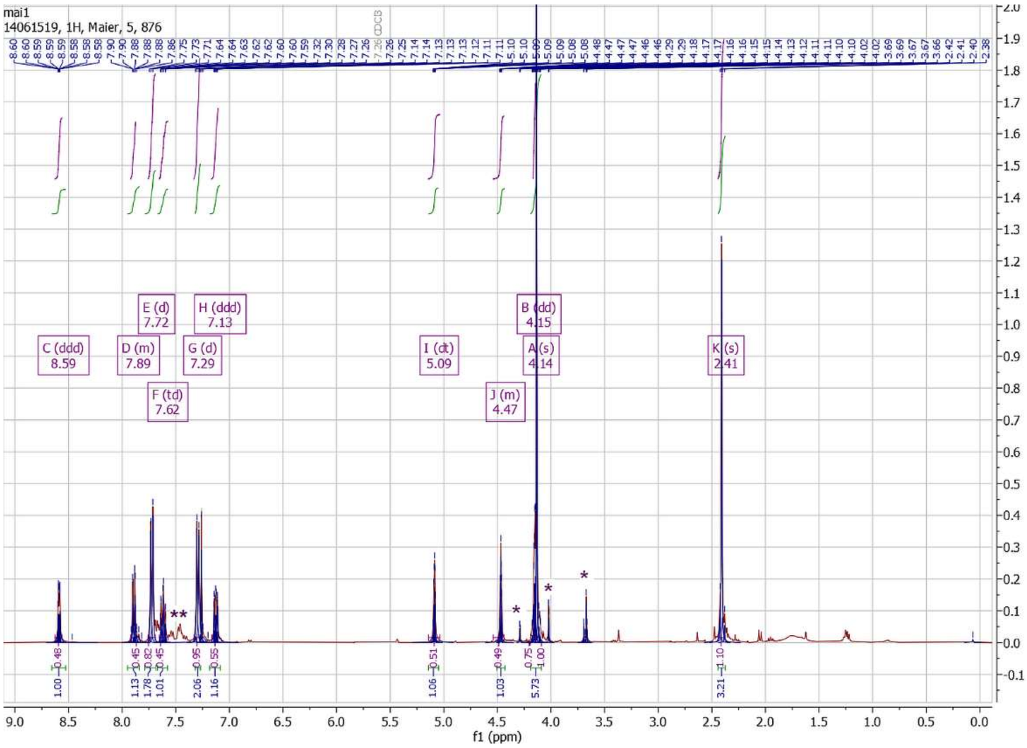

Figure S 1: ^{1}H NMR spectrum ($CDCl_3$, 400 MHz) of compound **3;** the signals marked with * are un-identified impurities

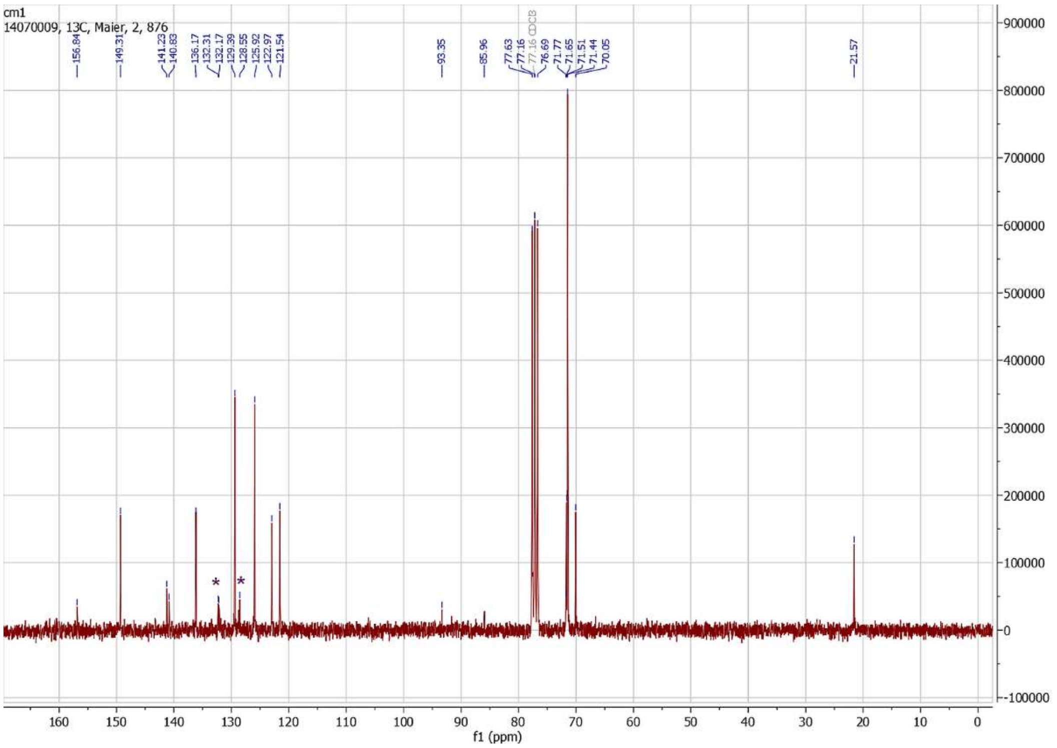

Figure S 2 $^{13}C\{^{1}H\}$ NMR spectrum ($CDCl_3$, 101 MHz) of compound **3;** The signals marked with * are un-identified impurities

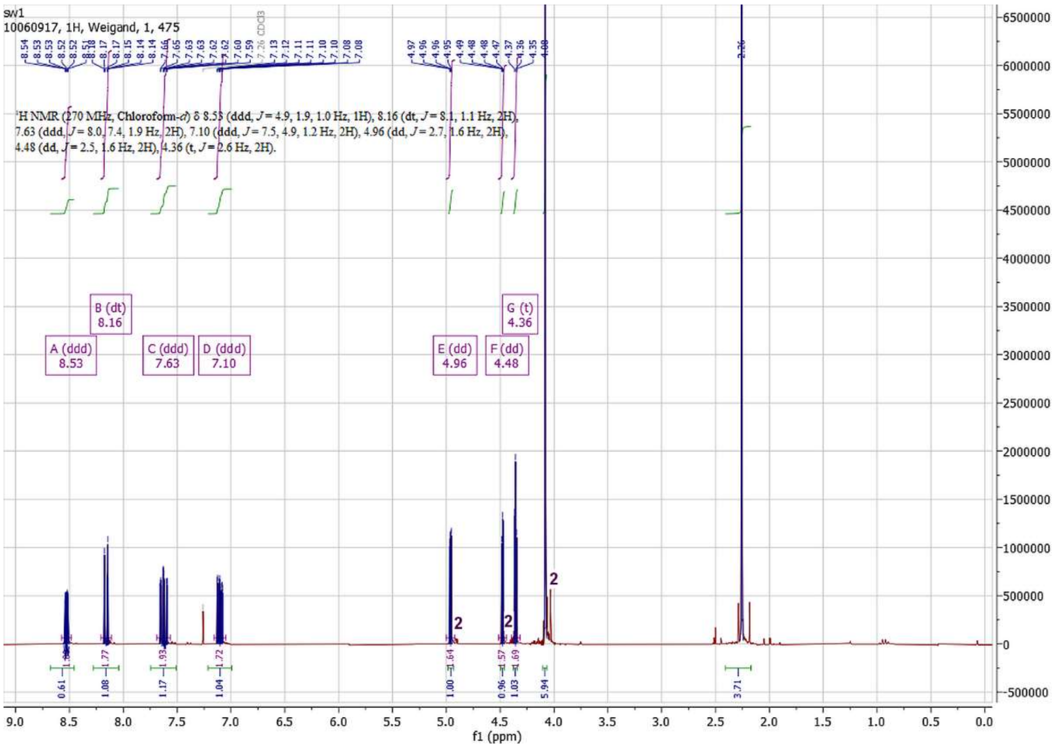

Figure S 3 ^{1}H NMR spectrum ($CDCl_3$, 270 MHz) of compound **5;** the signals marked with “2” belong to an impurity, which is most likely compound **2**

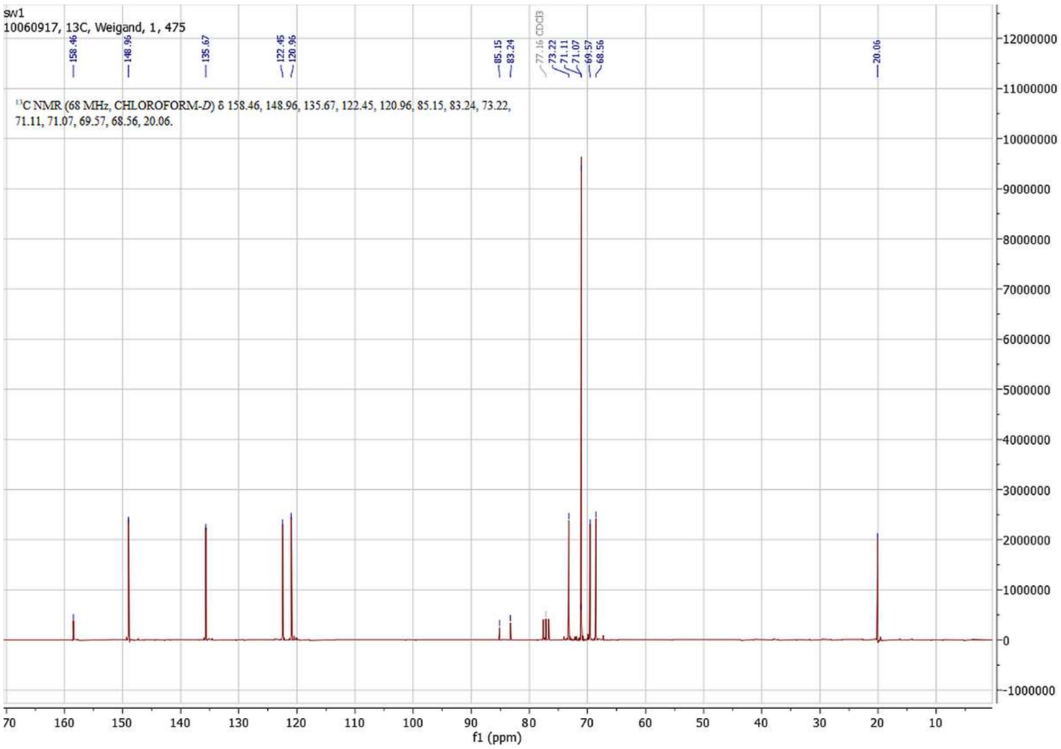

Figure S 4 $^{13}C\{^{1}H\}$ NMR spectrum ($CDCl_3$, 68 MHz) of compound **5**

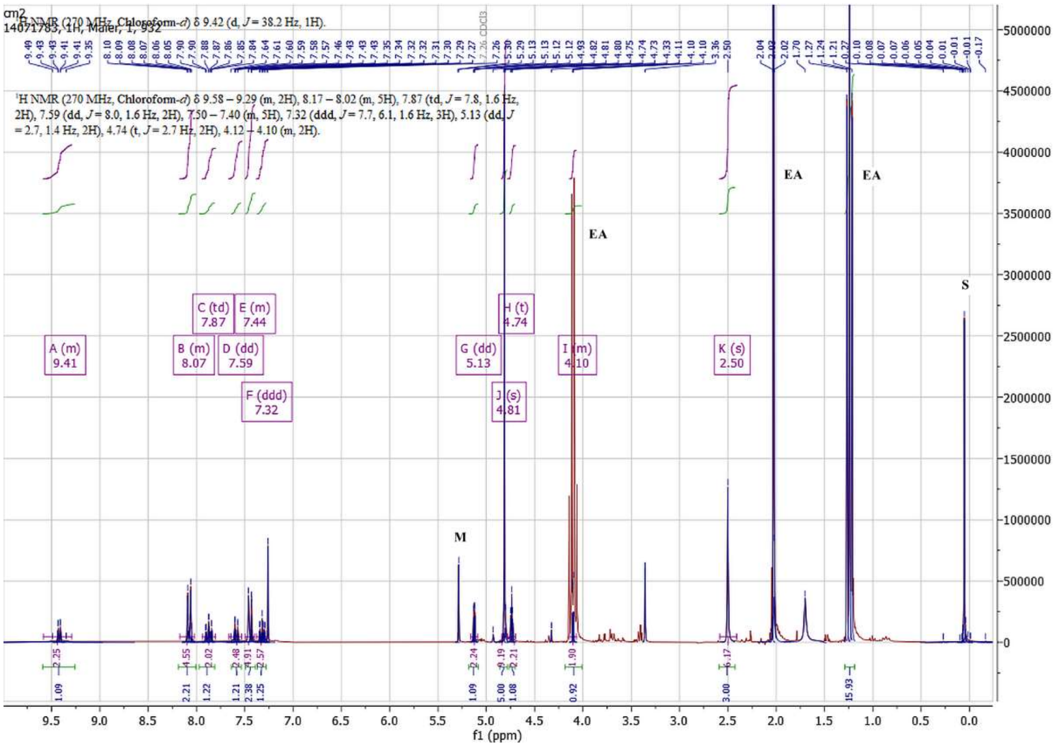

Figure S 5 ^{1}H NMR spectrum ($CDCl_3$, 270 MHz) of compound **4**

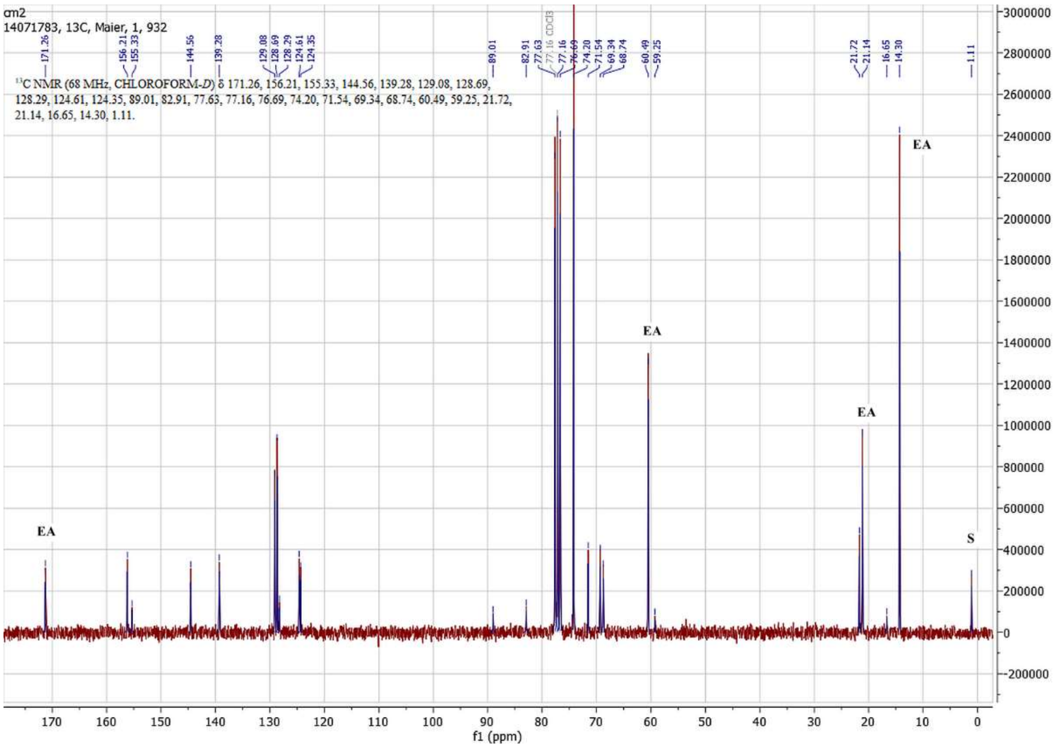

Figure S 6 $^{13}C\{^1H\}$ NMR spectrum ($CDCl_3$, 68 MHz) of compound **4**

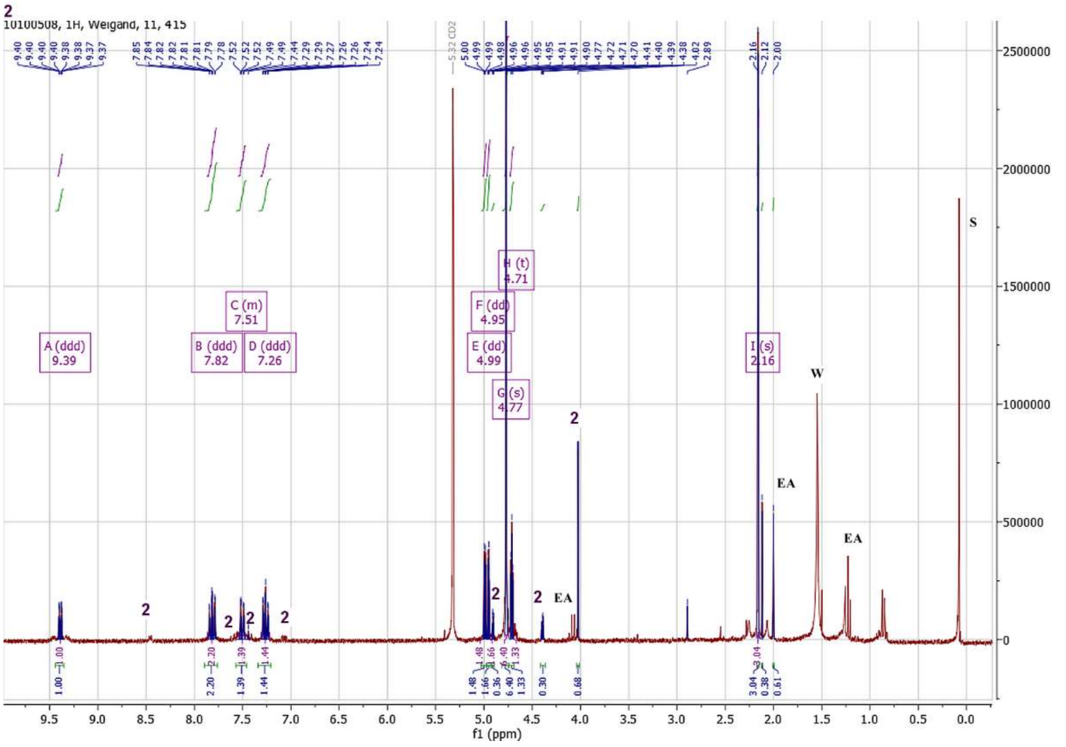

Figure S 7 ^{1}H NMR spectrum (CD_2Cl_2, 270 MHz) of compound **6;** the signals marked with “2” belong to an impurity, which is most likely compound **2**

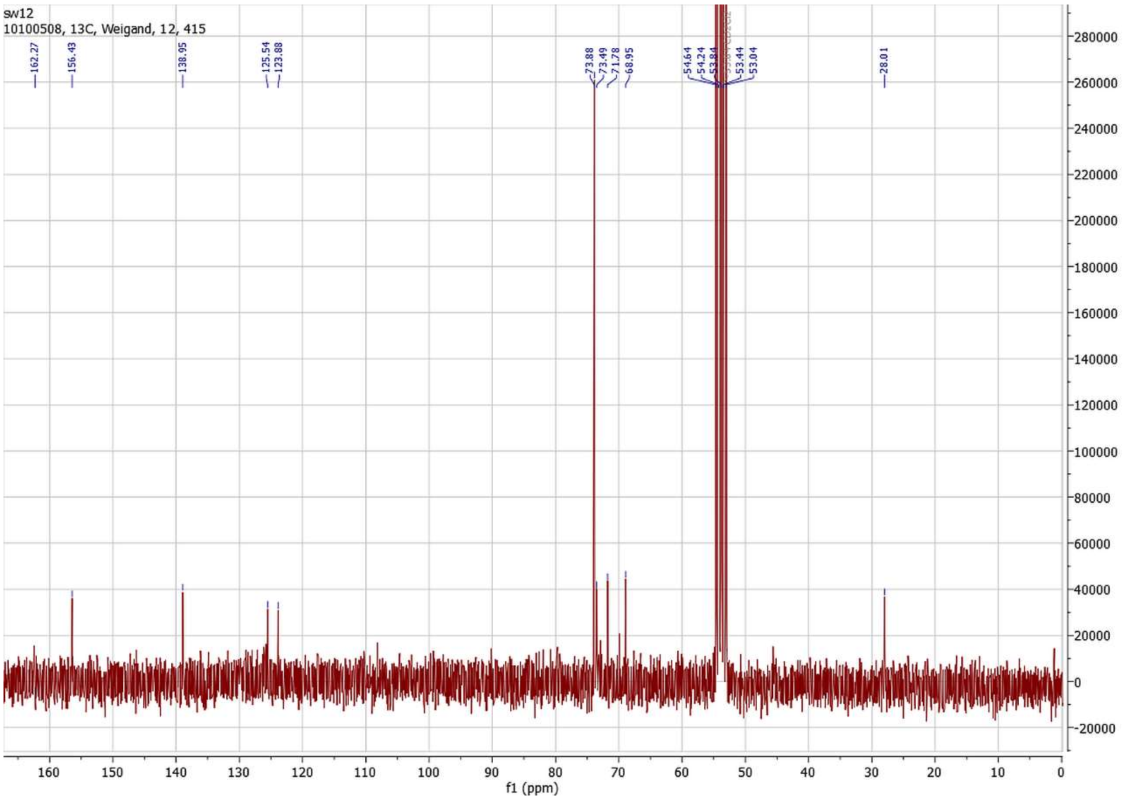

Figure S 8 $^{13}C\{^{1}H\}$ NMR spectrum (CD_2Cl_2, 68 MHz) of compound **6**

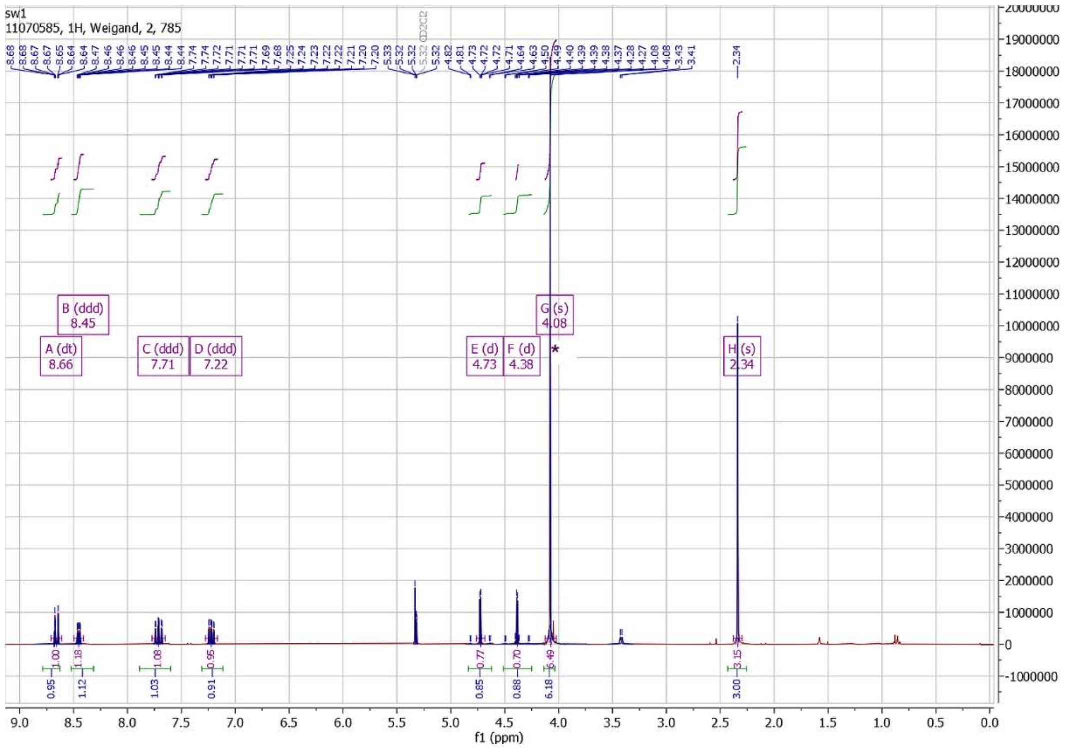

Figure S 9 ^{1}H NMR spectrum (CD_2Cl_2, 270 MHz) of compound **7**; * this signal is overlapping with a signal, that belongs to [Fe(C_5H_5){C_5H_3(C_5H_4N)(PtCl(Me_2SO))}]

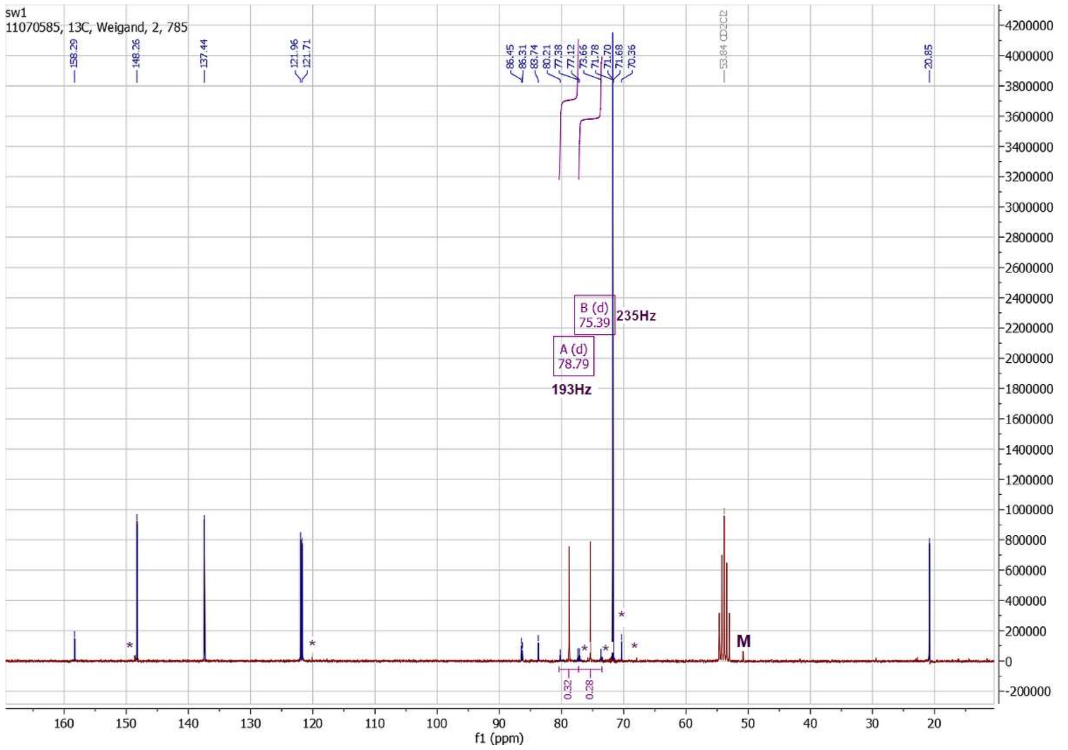

Figure S 10 $^{13}C\{^{1}H\}$ NMR spectrum (CD_2Cl_2, 68 MHz) of compound **7;** the signal marked with “M” belongs to a methylene dichloride impurity; the signals marked with an asterisk * belong to [Fe(C_5H_5){C_5H_3(C_5H_4N)(PtCl(Me_2SO))}]. The Cp signals at δ = 78.79 and 75.39 ppm have both Hg satellites with coupling constants *J*(C-Hg) of 193 Hz and 235 Hz, respectively
